# Effect of Azithromycin on Mineralized Nodule Formation in MC3T3-E1 Cells

**DOI:** 10.3390/cimb43030102

**Published:** 2021-10-06

**Authors:** Kengo Kato, Manami Ozaki, Kumiko Nakai, Maki Nagasaki, Junya Nakajima, Ryosuke Koshi, Hideki Tanaka, Takayuki Kawato, Morio Tonogi

**Affiliations:** 1 Department of Oral and Maxillofacial Surgery, Nihon University School of Dentistry, Tokyo 101-8310, Japan; deke18007@g.nihon-u.ac.jp (K.K.); nagasaki.maki@nihon-u.ac.jp (M.N.); deju20015@g.nihon-u.ac.jp (J.N.); tonogi.morio@nihon-u.ac.jp (M.T.); 2 Department of Oral Health Sciences, Nihon University School of Dentistry, Tokyo 101-8310, Japan; ozaki.manami@nihon-u.ac.jp (M.O.); nakai.kumiko10@nihon-u.ac.jp (K.N.); koushi.ryousuke@nihon-u.ac.jp (R.K.); tanaka.hideki@nihon-u.ac.jp (H.T.); 3 Division of Functional Morphology, Dental Research Center, Nihon University School of Dentistry, Tokyo 101-8310, Japan; 4 Division of Oral Structural and Functional Biology, Nihon University School of Dentistry, Tokyo 101-8310, Japan

**Keywords:** azithromycin, MC3T3-E1 cells, mineralized nodule, osteoblast, osteopontin

## Abstract

Azithromycin displays immunomodulatory and anti-inflammatory effects in addition to broad-spectrum antimicrobial activity and is used to treat inflammatory diseases, including respiratory and odontogenic infections. Few studies have reported the effect of azithromycin therapy on bone remodeling processes. The aim of this study was to examine the effects of azithromycin on the osteogenic function of osteoblasts using osteoblast-like MC3T3-E1 cells. Cells were cultured in the presence of 0, 0.1, 1, and 10 µg/mL azithromycin, and cell proliferation and alkaline phosphatase (ALPase) activity were determined. In vitro mineralized nodule formation was detected with alizarin red staining. The expression of collagenous and non-collagenous bone matrix protein was determined using real-time PCR or enzyme-linked immunosorbent assays. In cells cultured with 10 µg/mL azithromycin, the ALPase activity and mineralized nodule formation decreased, while the type I collagen, bone sialoprotein, osteocalcin, and osteopontin mRNA expression as well as osteopontin and phosphorylated osteopontin levels increased. These results suggest that a high azithromycin concentration (10 µg/mL) suppresses mineralized nodule formation by decreasing ALPase activity and increasing osteopontin production, whereas low concentrations (≤l.0 µg/mL) have no effect on osteogenic function in osteoblastic MC3T3-E1 cells.

## 1. Introduction

Azithromycin is a macrolide antibiotic that inhibits protein synthesis by binding to the bacterial 50S ribosomal subunit [1]. It displays immunomodulatory and anti-inflammatory effects in addition to broad-spectrum antimicrobial activity and is used to treat asthma and chronic obstructive pulmonary disease [2] as well as respiratory, urogenital, dermal, dental, and other bacterial infections [3,4]. It extensively penetrates tissues, and its concentrations are sustained after serum concentrations have declined to low levels [5]. Tonsillar azithromycin is maintained at higher concentrations than the minimum inhibitory concentration (MIC) required to inhibit pathogenic bacteria responsible for respiratory infections after multiple dose regimens (500 mg/day for 3 consecutive days or 500 mg/day for a day followed by 250 mg/day for 2–5 days) [6]. It is predicted that the azithromycin concentration in uterine or periodontal tissue is similarly maintained at high levels, despite antibiotic dose regimens dependent on the disease type and condition of the patients [6,7]. 

Osteoblasts and osteoclasts are involved in bone remodeling to maintain the mass and quality of osseous tissue [8]. Osteoblasts have osteogenic characteristics, including high alkaline phosphatase (ALPase) activity and production of bone matrix proteins, while osteoclasts secrete protons (H^+^) and proteases into the microresorptive area and decompose inorganic and organic bone tissue components [9]. Imbalanced osteoblast and osteoclast functions lead to osteoporosis and reduction in bone mineral density. The balance may be positively restored using bisphosphonate treatment to strongly inhibit osteoclastic bone resorption [10,11], whereas steroid therapy causes osteoblast apoptosis, which is an osteoporosis risk factor [12]. Some evidence exists that azithromycin stimulates alveolar bone regeneration in addition to its reduction in periodontal pathogens during administration to periodontal patients [13,14]. In vitro studies have indicated that azithromycin inhibits osteoclast differentiation and bone resorption activity in osteoclast procurer cells [15] and the production of inflammatory cytokines involved in bone metabolism in gingival fibroblasts [16]. Sub-antibiotic azithromycin doses attenuated alveolar bone destruction and improved trabecular microarchitectures in a rat model of experimental periodontitis [17]. The pre-existing periapical bone loss in a mouse model of periapical inflammation was also diminished by azithromycin administration [18]. These previous findings indicate that azithromycin may affect bone remodeling.

The aim of this study was to examine the effects of azithromycin on the osteogenic function of osteoblasts. Osteoblast-like MC3T3-E1 cells were continuously stimulated with azithromycin and examined for in vitro mineralized nodule formation, ALPase activity, and the expression of collagenous and non-collagenous bone matrix protein. 

## 2. Materials and Methods

### 2.1. Reagents 

Minimal essential medium α (MEMα) and heat-inactivated fetal bovine serum (FBS) were purchased from Gibco (Rockville, MD, USA) and HyClone Laboratories (Logan, UT, USA), respectively. Azithromycin, dimethyl sulfoxide (DMSO), and penicillin–streptomycin solution were obtained from Sigma (St. Louis, MO, USA).

### 2.2. Cell Culture and Azithromycin Stimulation

Murine osteoblastic MC3T3-E1 cells (ECACC 99072810, Culture collections, Public Health England, Salisbury, UK) were seeded on 100-millimeter culture dishes and maintained in MEMα containing 10% (*v*/*v*) FBS and 1% (*v*/*v*) penicillin–streptomycin solution at 37 °C in a humidified atmosphere of 95% air and 5% CO_2_. Cells were plated onto an appropriate culture plate at a density of 6.0 × 10^3^ cells/cm^2^, incubated overnight, then stimulated by the addition of 0.1, 1, or 10 µg/mL azithromycin (solubilized in DMSO), and further incubated for 10 or 14 days. Control cells contained a final concentration of 0.1% DMSO in the culture medium. The medium was changed every 2 days. 

### 2.3. Cell Proliferation and ALPase Activity

Cells were plated into 96-well plates at a density of 6.0 × 10^3^ cells/cm^2^ and cultured in medium with or without azithromycin for up to 10 days. Cell proliferation was determined using a cell-counting kit-8 (Dojindo Molecular Technologies, Inc., Kumamoto, Japan) according to the manufacturer’s protocols. ALPase activity was determined by adding 200 µL enzyme assay solution containing 8 mM *p*-nitrophenyl phosphate [19]. The absorbance was measured at 450 nm (cell counting) or 405 nm (*p*-nitrophenyl) using a microplate reader (SpectraMax^®^ ABS Plus; Molecular Devices LLC., San Jose, CA, USA). One unit of ALPase activity was defined as the amount of enzyme required for the formation of 1.0 µM *p*-nitrophenol per minute. Enzyme activity was recorded in milliunits (mU)/10^4^ cells.

### 2.4. Alizarin Red S Staining

Cells plated into 24-well plates at a density of 6.0 × 10^3^ cells/cm^2^ were cultured in medium with 50 mM β-glycerophosphate and 50 µg/mL ascorbic acid in the presence or absence of azithromycin for 14 days. The conditions of the cells and nodule formation were routinely checked via phase-contrast microscopy (Nikon DIAPHOTO ELWD 0.3, Tokyo, Japan) at 100× magnification. Mineralized nodules were detected by staining with alizarin red S (PG Research, Tokyo, Japan) according to a previous method [19]. After capturing images of the wells, 500 µL of 5% formic was added into the wells and incubated for 30 min. The solution was transferred to 96-well plates and the absorbance at 415 nm was determined for quantification.

### 2.5. Real-Time PCR

Cells were seeded into six-well plates at a density of 6.0 × 10^3^ cells/cm^2^ in medium with or without azithromycin for 10 days. Total RNA was isolated from cells harvested after a 7- or 10-day incubation using NucleoSpin^®^ RNA (Macherey-Nagel GmbH & Co., Düren, Germany). cDNA synthesis was conducted using 500 ng RNA in a total volume of 10 µL containing 50 pmol random 6 mers, 25 pmol origo dT Primer, 0.5 µL PrimeScript RT Enzyme Mix I, and 2 µL PrimeScript Buffer (these components were bundled with PrimeScript™ RT reagent Kit; Takara Bio Inc., Kusatsu, Japan). Aliquots of 2 µL of the cDNA solution were subjected to quantitative real-time PCR (RT-PCR). Reactions were performed in a total volume of 25 µL containing 2 µL cDNA solution, 12.5 µL TB green^®^ Premix Ex Taq II (Takara Bio Inc., Kusatsu, Japan), and 10 μM forward and reverse primers. Primer sequences were as follows: type I collagen (forward: 5′-TCAGTGCAATTGTGTTGCTGAAAG-3′; reverse: 5′-GATACCAAACTGGGCGTGCTG-3′), bone sialoprotein (forward: 5′-AATTCTGACCCTCGTAGCCTTCATA-3′; reverse: 5′-GAGCCTCGTGGCGACACTTA-3′), osteopontin (forward: 5′-TACGACCATGAGATTGGCAGTGA-3′; reverse: 5′-TATAGGATCTGGGTGCAGGCTGTAA-3′), osteocalcin (forward: 5′-CAGACACCATGAGGACCATCTT-3′; reverse: 5′-AAGGCTTTGTCAGACTCAGGG-3′), and glyceraldehyde 3-phosphate dehydrogenase (GAPDH forward: 5′-AAATGGTGAAGGTCGGTGTG-3′; reverse: 5′-TGAAGGGGTCGTTGATGG-3′). PCR was performed using a Thermal Cycler Dice real time system (Takara Bio Inc., Kusatsu, Japan) and analyzed using the instrument’s software. The cycling conditions reported in a previous study were used [11]. 

### 2.6. Enzyme-Linked Immunosorbent Assay (ELISA)

Culture supernatants were collected from plates on day 10. Phosphoprotein was purified using a phosphoprotein enrichment kit (Clontech Laboratories, Inc., Mountain View, CA, USA). Osteopontin levels in the crude and the phosphoprotein-purified sample were measured with an ELISA kit (R&D Systems, Minneapolis, MN, USA) according to the manufacturer’s protocols.

### 2.7. Statistical Analyses

Values are reported as means ± standard deviation. Significant differences were determined using a one-way analysis of variance followed by Tukey’s multiple comparison test. A *p*-value <0.05 was considered statistically significant. GraphPad Prism 6.0 software (San Diego, CA, USA) was used for statistical analyses.

## 3. Results

### 3.1. Effect of Azithromycin on Cellular Proliferation and ALPase Activity

**Figure 1:**
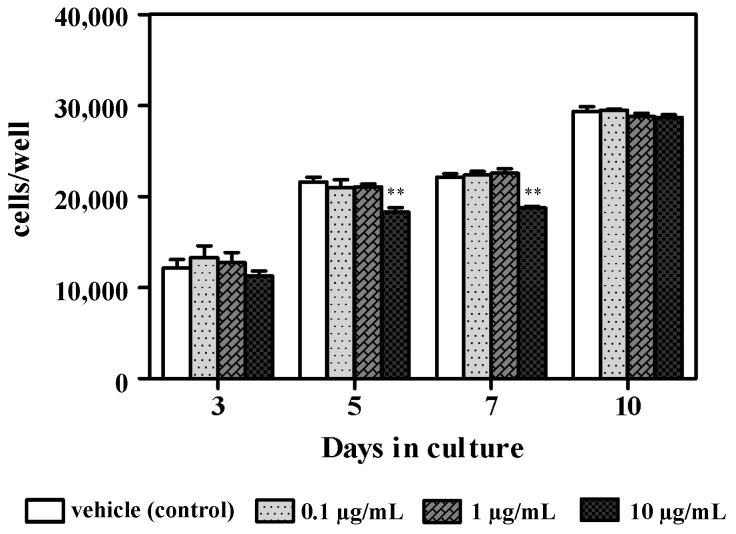
Effect of azithromycin on osteoblast proliferation. MC3T3-E1 cells were untreated (vehicle control) or grown in the presence of variable azithromycin concentrations (0.1, 1, or 10 µg/mL) for 10 days. Data represent the mean ± SD of three independent experiments. ** *p* < 0.01 compared with the control.

**Figure 2:**
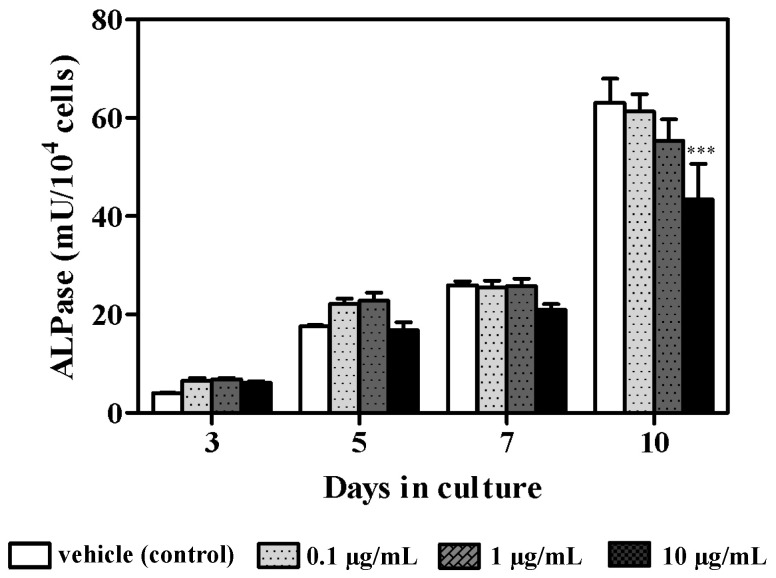
Effect of azithromycin treatment on ALPase activity. MC3T3-E1 cells were untreated (vehicle control) or grown in the presence of variable azithromycin concentrations (0.1, 1, or 10 µg/mL) for 10 days. Data represent the mean ± SD of three independent experiments. *** *p* < 0.001 compared with the control.

Azithromycin concentrations of 0.1 and 1 µg/mL did not affect osteoblast cell proliferation at all time points, whereas significantly decreased growth was observed on days 5 and 7 following treatment with 10 µg/mL azithromycin compared with untreated cells (Figure 1). There was no difference in cell proliferation at all azithromycin concentrations on day 10. Meanwhile, ALPase activity gradually increased in untreated cells and azithromycin-stimulated cells during the culture period (Figure 2). ALPase activity significantly decreased following treatment with 10 µg/mL azithromycin on day 10 compared with the untreated control (Figure 2). 

### 3.2. Effect of Azithromycin on Mineralized Nodule Formation 

**Figure 3:**
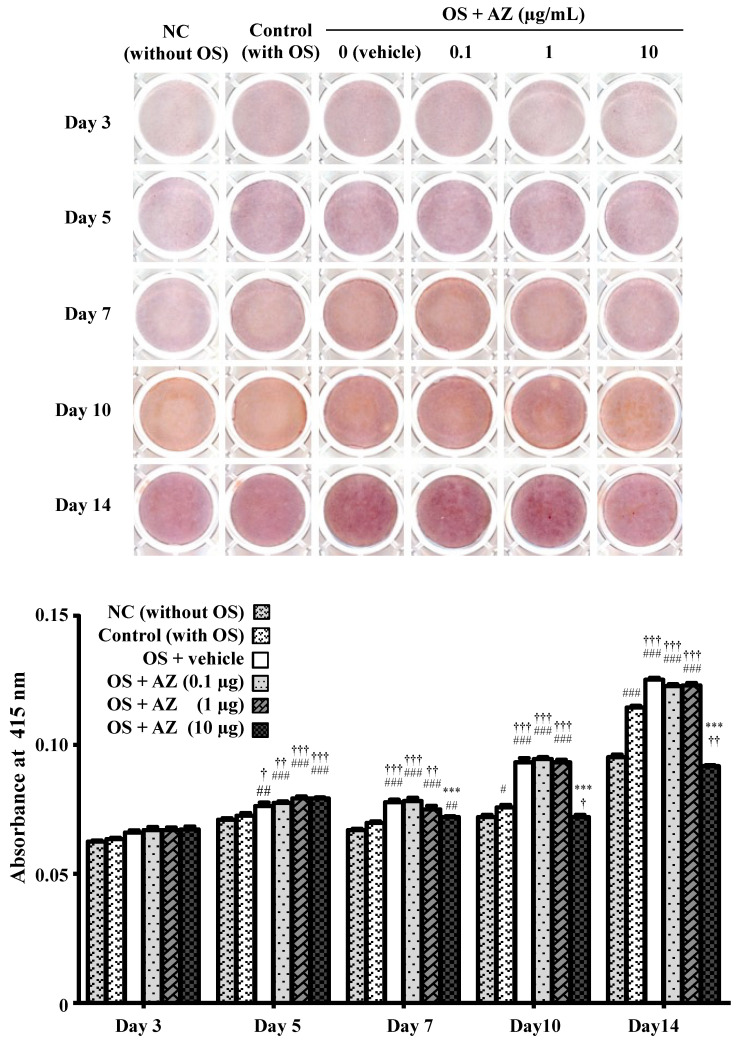
Effect of azithromycin on mineralized nodule formation. MC3T3-E1 cells were treated with 0.1, 1, or 10 µg/mL azithromycin in the presence of osteogenic supplements (OS; 50 mM β-glycerophosphate and 50 µg/mL ascorbic acid) and 0.1% DMSO as a vehicle. Mineralized nodule formation was examined by alizarin red staining. Data represent the mean ± SD of three independent experiments. NC, negative control. ^###^ *p* < 0.001, ^##^ *p* < 0.01, ^#^ *p* < 0.05 compared with negative control (NC); ^†††^ *p* < 0.001, ^††^ *p* < 0.01, ^†^ *p* < 0.05 compared with control; *** *p* < 0.001 compared with the vehicle control.

A previous study reported that DMSO at a concentration of 0.2% or less had no effects, whereas DMSO at a concentration of 0.5% or more increased osteogenic function in MC3T3-E1 cells [20]. Our pilot study indicated that 0.1% DMSO slightly increased the expression of Runx2, an osteoblast differentiation-related factor, in MC3T3-E1 cells (data not shown); therefore, we examined the effects of azithromycin on mineralized nodule formation in the presence of osteogenic supplements (OS; 50 mM β-glycerophosphate and 50 µg/mL ascorbic acid) and 0.1% DMSO as a vehicle (Figure 3). The intensity of alizarin red staining increased in the control (with OS) and the vehicle control compared with the negative control (NC) without OS. Azithromycin reduced staining intensity at a concentration of 10 µg/mL compared with the vehicle control and control (with OS).

### 3.3. Effects of Azithromycin on Bone Matrix Protein mRNA Expression

**Figure 4:**
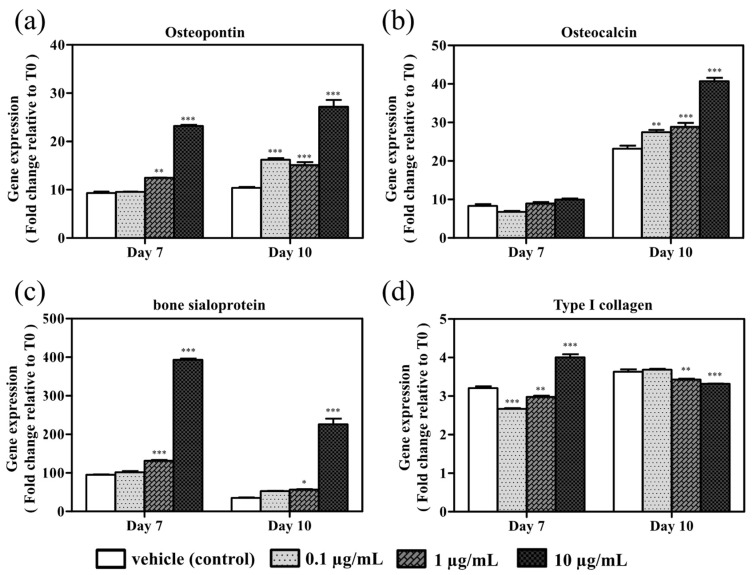
Effect of azithromycin on (**a**) osteopontin, (**b**) osteocalcin, (**c**) bone sialoprotein, and (**d**) type I collagen mRNA expression determined by real-time PCR following 7- and 10-day culture. MC3T3-E1 cells were untreated (vehicle control) or grown in the presence of variable azithromycin concentrations (0.1, 1, or 10 µg/mL). The expression level of each gene was calculated and expressed as a ratio to the expression level in cells without azithromycin and DMSO treatment on the day when cells were seeded (T0). Data represent the mean ± SD of three independent experiments. * *p* <0.05, ** *p* < 0.01, and *** *p* < 0.001 compared with the vehicle control on each day of culture.

The osteopontin, osteocalcin, bone sialoprotein, and type I collagen mRNAs were differentially expressed to variable degrees after treatment for 7 and/or 10 days in cells stimulated with azithromycin at 0.1, 1, and 10 µg/mL compared with untreated control cells (Figure 4a–d). Osteopontin and bone sialoprotein expression on days 7 and 10 of culture increased with application of 0.1, 1 or 10 µg/mL azithromycin compared with the untreated control (Figure 4a,c). These concentrations of azithromycin induced osteocalcin expression on day 10 of culture (Figure 4b). The greatest differences in expression were observed at 10 µg/mL azithromycin. Type I collagen expression increased on day 7 following treatment with 10 µg/mL azithromycin compared with the untreated control, whereas a decrease was observed with azithromycin treatment at 0.1 and 1 µg/mL. The expression of type I collagen significantly decreased on day 10 following azithromycin treatment at 1 and 10 µg/mL compared with the control (Figure 4d).

### 3.4. Effect of Azithromycin on Osteopontin Levels

**Figure 5:**
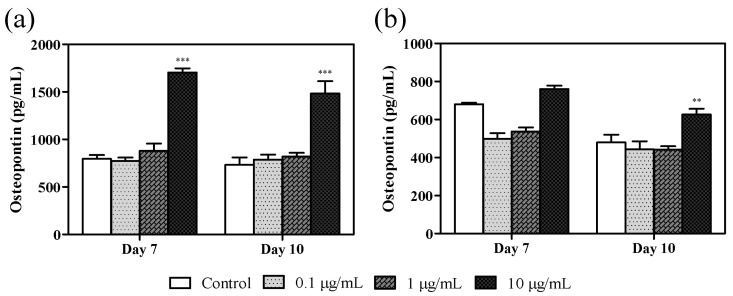
Effect of azithromycin on osteopontin levels. MC3T3-E1 cells were untreated (vehicle control) or grown in the presence of variable azithromycin concentrations as stated in the legend. The levels of (**a**) osteopontin in the supernatant and (**b**) phosphorylated osteopontin in the phosphoprotein-purified supernatant were determined using enzyme-linked immunosorbent assays 7 and 10 days after treatment. Data represent the mean ± SD of three independent experiments. ** *p* < 0.01 and *** *p* < 0.001 compared with the vehicle control.

Osteopontin levels significantly increased in the crude culture supernatant derived from cells stimulated with 10 µg/mL azithromycin after 7 and 10 days following treatment compared with the untreated control (Figure 5a). Phosphorylated osteopontin levels increased in cells stimulated with 10 µg/mL azithromycin on days 7 and 10 compared with the control, with a significant difference observed on day 10 (Figure 5b). 

## 4. Discussion

Several previous studies reported sustained azithromycin concentration in periodontal tissue following its administration to periodontitis patients [7,21,22,23]. The clinical efficacy of azithromycin has been demonstrated in odontogenic infections characteristic of gingiva, periodontal ligament, and alveolar bone destruction [4,13,14]. Malizia et al. reported a higher azithromycin concentration in the periodontal tissue than in the plasma of patients who were orally administrated azithromycin; the concentration of azithromycin in the gingiva, alveolar bone, and plasma was 6.47 ± 0.57 mg/kg, 1.86 ± 0.15 mg/kg, and 0.33 ± 0.04 mg/L, respectively, in these patients 12 h following its administration at a dose of 500 mg/day for 3 consecutive days, with a gradual decline observed over several days [21]. There is no marked difference in the prescription between periodontitis [21] and respiratory infection [6]. In the present study, there was no significant difference in mineralized nodule formation by MC3T3-E1 cells treated with azithromycin at concentrations ≤1 µg/mL compared with the control. These findings and our results indicate that ordinary dose regimens of azithromycin for the treatment of respiratory and odontogenic infections are unlikely to influence osteogenesis by osteoblasts. Other clinical studies reported the bone regenerative potential of azithromycin in patients with bone destruction caused by periodontal abscesses [13,14]. Moreover, azithromycin concentrations below 10 mg/mL markedly suppressed osteoclast bone resorption in vitro [15]. Azithromycin at concentrations ranging from 0.1 to 10 µg/mL attenuates lipopolysaccharide (LPS)-induced production of pro-inflammatory cytokines, including interleukin 6 which associates with osteoclast differentiation [16]. These findings and our present results suggest that potential bone regeneration induced by azithromycin may be associated with the downregulation of osteoclastic bone resorption and not the upregulation of osteoblastic bone formation. 

Furthermore, in this study, ALPase activity and mineralized nodule formation in MC3T3-E1 cells were markedly suppressed with 10 µg/mL azithromycin, whereas mRNA expression of type I collagen, bone sialoprotein, osteocalcin, and osteopontin increased. Type I collagen is a crucial scaffold, while bone sialoprotein and osteocalcin are indispensable for the initiation of bone mineralization [24,25,26]. The present results show that increased collagenous and non-collagenous protein expression does not contribute to mineralized nodule formation when there is decreased ALPase activity. Moreover, the role of osteopontin in calcification and the interaction of ALPase, pyrophosphate, and osteopontin may explain the suppression of mineralized nodule formation in cells cultured with 10 µg/mL azithromycin. ALPase hydrolyzes pyrophosphate, which has an inhibitory effect on hydroxyapatite crystal growth [8,27], and pyrophosphate stimulates osteopontin production in MC3T3-E1 cells [28]. Moreover, phosphorylated osteopontin inhibits hydroxyapatite formation [28,29], whereas ALPase attenuates this inhibitory effect [29,30,31]. In the present study, osteopontin and phosphorylated osteopontin levels increased following treatment with 10 µg/mL azithromycin, whereas ALPase activity markedly decreased. Therefore, the high azithromycin concentration (10 µg/mL) suppressed mineralized nodule formation by increasing phosphorylated osteopontin production and decreasing ALPase activity.

It is well known that azithromycin tends to accumulate in inflamed tissues [1,2,3]. Blandizzi et al. reported that azithromycin levels were significantly higher in pathological tissue, reaching a concentration of approximately 10 mg/kg in marginal periodontitis, periapical periodontitis, radicular granuloma, and the cyst wall of dentigerous cyst compared with that in normal gingiva 2.5 days after oral administration of 500 mg azithromycin/day for 3 consecutive days [22]. Accumulation of azithromycin in tissues surrounding the bone may inhibit osteoblastic bone formation following frequent or long-term administration of the drug.

## 5. Conclusions

High azithromycin concentration (10 µg/mL) suppressed mineralized nodule formation by decreasing ALPase activity and increasing osteopontin production, whereas low concentrations (≤l.0 µg/mL) had no effect on osteogenic function in osteoblastic MC3T3-E1 cells.

## Data Availability

Available data are presented in the manuscript.
